# Livelihood Diversification in Tropical Coastal Communities: A Network-Based Approach to Analyzing ‘Livelihood Landscapes’

**DOI:** 10.1371/journal.pone.0011999

**Published:** 2010-08-11

**Authors:** Joshua E. Cinner, Örjan Bodin

**Affiliations:** 1 Australian Research Council Centre of Excellence for Coral Reef Studies, James Cook University, Townsville, Queensland, Australia; 2 Stockholm Resilience Centre, Stockholm University, Stockholm, Sweden; 3 Department of Systems Ecology, Stockholm University, Stockholm, Sweden; Stanford University, United States of America

## Abstract

**Background:**

Diverse livelihood portfolios are frequently viewed as a critical component of household economies in developing countries. Within the context of natural resources governance in particular, the capacity of individual households to engage in multiple occupations has been shown to influence important issues such as whether fishers would exit a declining fishery, how people react to policy, the types of resource management systems that may be applicable, and other decisions about natural resource use.

**Methodology/Principal Findings:**

This paper uses network analysis to provide a novel methodological framework for detailed systemic analysis of household livelihood portfolios. Paying particular attention to the role of natural resource-based occupations such as fisheries, we use network analyses to map occupations and their interrelationships- what we refer to as ‘livelihood landscapes’. This network approach allows for the visualization of complex information about dependence on natural resources that can be aggregated at different scales. We then examine how the role of natural resource-based occupations changes along spectra of socioeconomic development and population density in 27 communities in 5 western Indian Ocean countries. Network statistics, including in- and out-degree centrality, the density of the network, and the level of network centralization are compared along a multivariate index of community-level socioeconomic development and a gradient of human population density. The combination of network analyses suggests an increase in household-level specialization with development for most occupational sectors, including fishing and farming, but that at the community-level, economies remained diversified.

**Conclusions/Significance:**

The novel modeling approach introduced here provides for various types of livelihood portfolio analyses at different scales of social aggregation. Our livelihood landscapes approach provides insights into communities' dependencies and usages of natural resources, and shows how patterns of occupational interrelationships relate to socioeconomic development and population density. A key question for future analysis is how the reduction of household occupational diversity, but maintenance of community diversity we see with increasing socioeconomic development influences key aspects of societies' vulnerability to environmental change or disasters.

## Introduction

Livelihoods in tropical coastal communities often rely on a range of occupational sectors, such as agriculture, fisheries, and informal economic activities (i.e. small shops, transportation, etc.) [1]–[4]. Examining how households access, and depend upon a diversity of occupational sectors is a central theme in many development studies and is often discussed in the context of poverty, urbanization, household risk, conservation, and coping strategies [4]–[8]. Several frameworks have been developed for examining coastal livelihoods, which emphasize connections and interdependence between fisheries and other occupational sectors [2], [3]. However, these frameworks lack a way to examine system-level measures of the whole set of occupations and their interrelations, what we refer to as the ‘livelihood landscape’. Furthermore, we also perceive a need for a comprehensive and readily understandable way to capture and illustrate these often quite complicated livelihood landscapes. In this article, we develop a novel methodological framework to provide insights into the role of key natural resource-based sectors (such as fisheries) in the wider economy.

Quantitative approaches such as social network analyses are becoming increasingly utilized to help scientists and managers better understand social phenomena in a wide variety of disciplines from psychology to economics including interdisciplinary fields such as natural resource management [9], [10]. In this context, we developed and applied a novel network modeling approach to illustrate and analyze the patterns of occupational dependencies and their interrelationships at different levels of social aggregation (e.g., village, regional or country levels). Key advantages to using network analysis to examine livelihoods are that it provides measures on how each occupation relates to the other occupations as well as it enables systemic measures of the livelihood landscape. Here, we studied in- and out-degree centrality of individual occupations, and network density and centralization; corresponding to sectoral and systemic types of analyses respectively. When used in a livelihood analysis, these measures of centrality, density and centralization provide novel information about the relative importance of specific sectors such as fisheries, which along with information on patterns of occupational interrelationships, can be quantitatively examined and compared.

We use this novel network-based livelihoods approach to further explore an empirical observation from a related paper about the relationship between natural resource use and socioeconomic development in coastal societies [11]. Sociological perspectives on human-environment interactions suggest that socioeconomic development can have profound influences in how societies use local natural resources [12]–[14]. Importantly, though, it is generally not considered that the level of socioeconomic development, per se, impacts resource conditions directly, but rather that there tend to be accompanying changes in the composition of the economy, the technologies people use, and also an increased scale at which wealthier societies are able to extract resources [15]. For example, in the aforementioned related study, observation of a Kuznets-like (i.e. U-shaped) relationship between the biomass of coral reef fishes and socioeconomic development in the western Indian Ocean (WIO) was partially explained by statistical differences in the proportion of households involved in select occupations, the types of gears used, and the use of boats with engines between low, medium, and high development sites [11]. In this present paper, we use the livelihood landscapes approach to dig deeper into the former of these potential explanations; the so-called composition effect, whereby development is expected to be associated with a changing composition of local economies from natural resource extraction to sectors which may be less destructive to the local environment, such as a service economy [16]. According to this perspective, one would expect that that the importance of natural resource-based occupations would decrease with development, and that the importance of other sectors such as salaried employment and tourism would increase with development.

We also examine the relationship between human population density and peoples’ livelihood portfolios [4], [17]. Human population density has been related to livelihood strategies in places as diverse as Latin America, Melanesia, and Africa [4], [18]–[20]. In the context of rural economies, high population density can potentially reflect land constraints (such as land fragmentation) [20], influence land to labor ratios which may affect the profitability of certain livelihood strategies, and can create comparative advantages for certain types of occupations, for example by providing ready markets for products [18].

Our overarching research questions are: “how are different economic sectors connected in the context of household economies in tropical coastal communities?” and “how do key occupational sectors and livelihood landscapes in coastal communities change along spectra of socioeconomic development and population density?” To address these questions, we first use a network approach to represent livelihoods as ‘landscapes’. In doing so, we provide a new framework for detailed systemic analysis of household livelihood portfolios which can be examined at varying scales of social aggregation. Specifically, we examine livelihood landscapes in Kenya at: (1) a single peri-urban community; (2) at an aggregate of peri-urban communities; (3) at an aggregate of rural communities (to provide a rural-urban contrast); and (4) at a ‘national’ aggregate of all rural and peri-urban Kenyan communities studied. We explore livelihood landscapes at these differing scales to illustrate the role of key sectors such as fisheries in the context of the wider economy. We then explore the composition effect by comparing network measures of centrality, centralization, and density with a multi-variate index of socioeconomic development and measures of human population density from 27 communities across Kenya, Tanzania, Madagascar, Seychelles, and Mauritius. Specifically, we ask the following questions: (1) “Do key characteristics of livelihood landscapes vary predictably with the level of socioeconomic development or population density?” and (2) “How does the position of each occupation in the ‘livelihood landscape’ relate to the degree of development and population density?”

## Methods

### Study sites

We studied 27 coastal communities in Kenya, Tanzania, Madagascar, Seychelles, and Mauritius. Sites were purposively selected as part of a larger project linking social and ecological systems across the western Indian Ocean (WIO) [11], [21], [22]. We surveyed a total of 1564 households. Sampling of households within communities was based on a systematic sampling design [23]. We conducted between 23–143 surveys per site, depending on the population of the communities and the available time per site. This represented between 10–66% of the households in a community. A household was defined as people living together and sharing meals.

### Collecting data on occupations

We examined dependence on fishing and other livelihood activities by asking respondents to list all of the jobs people in the household engaged in for food or money. We grouped occupations into the following categories: fishing, selling marine products, tourism, farming, cash crops, gleaning, salaried employment, the informal sector, other, and ‘none’. Gleaning is the collection of marine organisms from shallow or intertidal areas and generally focuses on octopus and sea cucumbers. The informal sector is comprised of casual labor or entrepreneurial activities that tend to provide daily compensation with no benefits (e.g. health insurance or annual leave). Across all countries, the most common jobs in this sector were: independent tradesman work (construction, plumbing, painting, masonry), selling food (e.g. a produce stand), small shop or kiosk ownership, and quarry work. Salaried employment was employment such as government work, which resulted in a regular salary. The ‘other’ sector comprised of activities such as being a traditional healer or receiving remittances. When multiple occupations were present in a single household (which was almost always the case), we then asked respondents to rank these activities in order of importance. Thus, respondents would define which occupation was primary, secondary, tertiary, etc.

### Socioeconomic development index

Community leader interviews were used to determine the presence or absence of the following community-level infrastructure items [adapted from Pollnac [24]]: hospital, medical clinic, doctor, dentist, primary school, secondary school, piped water, sewer, sewage treatment, septic tanks, electricity service, phone service, food market, pharmacy, hotel, restaurant, petrol station, public transportation, paved road, banking facilities. These items were then combined into an index of community-level socioeconomic development using a Principle Component Analysis, as reported in [21].

### Human population density

Population density data was collected using the Socioeconomic Data and Applications Center (SEDAC) gridded population of the world database (available Online http://sedac.ciesin.org/gpw/global.jsp). Geographic coordinates of field sites were overlaid on the gridded population database. Grid cells were 4.66 km^2^. When a field site was near the border of two grids, those grids were averaged to give a mean population density. Population density was transformed using the natural log function. Population density was only available for 25 sites because two sites were on islands too small to be picked up on the global population database.

### Representing ‘livelihood landscapes’ in coastal communities

**Figure 1:**
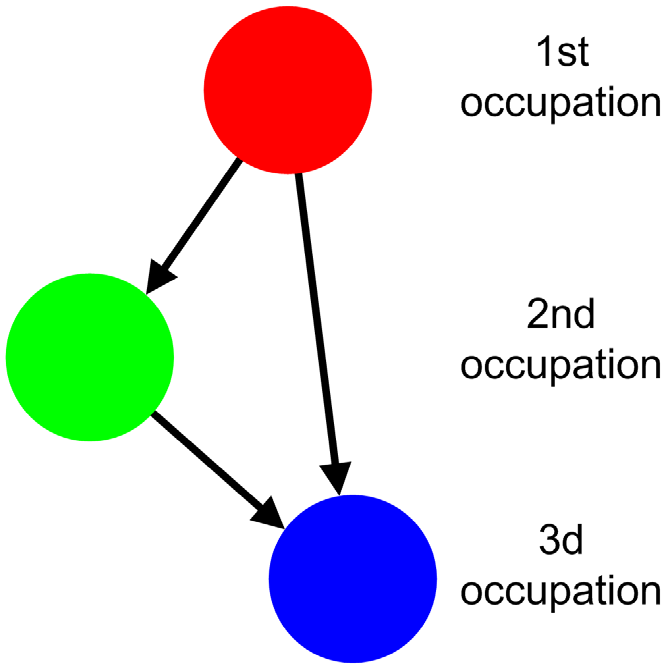
A heuristic model of a livelihood network for a single household. The nodes represent different occupations conducted by the members of the household. Here, the household has three different occupations (first, second and third occupation represented by the three nodes in different colors). The resulting links are directed according the ranked order of the occupations.

The first step in analyzing the livelihood landscape is to construct a map encompassing all occupations and their pattern of interrelations for a chosen set of households (the respondents). In this study, we modeled different livelihood landscapes as networks where the nodes represent different occupations, and where links between pairs of nodes represent respondents who have reported both the corresponding occupations. All the links are directed, pointing from the higher-ranked to the lower-ranked occupations. Hence, a household that has specified fishing as their primary occupation, and farming as their secondary occupation, will be represented by a link going from the primary node (representing fishing) to the secondary node (representing farming). Furthermore, if a household has reported a third occupation, links are created both from the main and the secondary occupational nodes in the network (Fig. 1). For each node we recorded the number of households who reported the corresponding occupation, and for each link we recorded how many households that constituted that link. The resulting maps will then topologically represent, as networks, the livelihood landscape constituting all realized occupations and their pattern of interrelations.

Schematically, these network maps were created according to the following procedure:

1. Create a list of all households given some selection criteria (e.g., all households in a particular village).
2. Create one node for each different occupation (e.g. fishing, farming, etc.) among the sampled households.
3. Assign a primary counter to each node, and set that counter to zero.
4. Assign a secondary counter to each node and set that counter to zero as well.
5. For each household in the sample, do the following:
  1. Increment the primary counter by one for the specific node corresponding to the main occupation reported by that household.
  2. Increment the secondary counters by one for all nodes corresponding to all of the less important occupations reported by that household.
  3. Create (directed) links between these occupations and assign a counter to each link. Set the counter to one. If links already exist, instead increment the corresponding link counters by one.

This procedure not only produces a topological network map of the livelihood landscape, it also assigns weights to each occupational interrelationship (i.e. link). The weights equal the number of households that constitutes the links (i.e. the values of the link counters described above). To make link weights more comparable, we normalized them by dividing the value of the link counter with the value of the secondary counter of the originating node. Hence, the normalized link weights correspond to the fraction of the households that reported the occupation represented by the originating node that also reported the occupation of the destination node, but the latter with a lower ranking. In this study, our focus is on the household scale because we consider households, rather than individuals, to be the most relevant economic unit and the appropriate scale at which occupational dependencies arise. Accordingly, this normalization procedure only accounts for the fraction of *households* that have reported these occupational relationships and it does not account for the fraction of *individuals*. Therefore, the strength of the interdependency between any two occupations depends only on the number of households that are engaged in both, and not on the number of individuals in these households (which typically varies between households). For comparative purposes, however, we have also normalized the link strength based on the number of individuals engaged in each occupation, and these results we present separately in supplemental material.

Technically, this approach of creating a network of occupational interdependencies draws from the 2-mode network approach (where one type of nodes represents ‘events’, and the other type of nodes represents actors visiting these events [for an overview of different kinds of network centralities, see 25]). Such 2-mode network (often referred to as affiliation network) can easily be converted to a 1-mode network that only consists of events, and where links between events are representing actors visiting both events [25]. In our case, occupations represent events, households are the actors engaged in these events, and the 1-mode livelihood landscapes are the converted 2-mode networks of occupations and households. However, we have also taken the ranking of the different occupations into account, thus all links between the different occupations are, in our case, weighted separately for each direction. Hence, the resulting network not only shows interdependencies between occupations (there being links between different occupations), it also shows to what extent two different occupations are perceived as being more or less important vis-à-vis each other (the directionally of the links), and how large proportion of the households being engaged in any pair of occupations (the link weights).

### Interpreting and analyzing the livelihood networks

The resulting networks were drawn in order to graphically illustrate the complicated patterns of occupational dependencies for the different aggregates of households. We used the computer program NetDraw, which is part of the Ucinet software package [26], to make the drawings. For improved readability, all the links where less than 5% of the households who reported the occupation of the originating node also reported the occupation of the destination node (with a lower rank) were omitted from the maps of the livelihood landscapes. To add additional information (such as the number of respondents who listed a node as a primary occupation or as a lower ranking occupation), the drawings were also manually refined so that the size of each node corresponded to the number of respondents who had reported the corresponding occupation. Additionally, the proportion of respondent that had reported the occupation as their primary source of income was visually illustrated by representing the node as a pie chart. Likewise, the thickness of the lines connecting different nodes corresponded to the weight of underlying links. Finally, the positions of the nodes were determined using spring-embedded layout techniques [26]. In using these layout techniques, the position of the nodes in the plot is determined by their composition of links to the other nodes. Hence, a node positioned in the middle of the drawing is thus, to some extent, linked to all other nodes in the network in a uniform manner. That means it is positioned in the network in balanced way implying that it is equally connected to all other. Hence, a node located in the middle of the figure represents an occupation engaged in by households that also engage in other occupations without any strong commonly shared propensities for any particular other occupation. In the same way, a node in the periphery of the figure represents an occupation where the respondents, if they also did report other occupations, tended to pick these from a limited set of occupations. The level to which a particular occupation occupies a position close to the centre of such a plot is from now on referred to as its level of *uniform embeddedness* and should be distinguished from its level of centrality (discussed below).

In addition to the network maps of livelihood landscapes which qualitatively demonstrate the underlying patterns of occupational interrelations, we were also interested in using formal quantitative analyses of the livelihood landscapes with the objective to capture and assess several structural characteristics that might be of relevance in explaining socioeconomic development in communities. Here, we divide these kinds of analyses in two categories; one category where the focus is on a particular occupational sector (e.g. fishing) and how it relates to the other occupations in the community, and the second category where the focus is on the complete pattern of all occupational interrelations. The latter is a systemic analysis.

#### i) Analysis of individual occupations

Perhaps the most obvious characteristics that comes to mind when looking at a particular occupation in the livelihood landscape is how many links that goes to or from it. If it has many links, the households that have listed this particular occupation have also listed a high number of other occupations. Hence, the number of links associated with a particular occupation defines how many other occupations the corresponding households are associated with. From a socio-economic perspective this is interesting to know since it shows how households differentiate their incomes among different combinations of occupations.

In network terminology, the number of links associated with a particular node is defined as the node's *degree centrality* [27]. Furthermore, in this study the links are directed, hence one differentiates between *in-degree* and *out-degree* centrality (incoming versus outgoing links respectively). Hence, a node's in-degree centrality represent to what extent the occupation is chosen as a lower-ranked occupation (and vice versa for the out-degree centrality). A node with low scores on both in-degree and out-degree centrality is thus normally chosen as the main and only occupation, whereas nodes with higher scores of in- and/or out-degree centralities are often chosen in combination with other occupations.

The in- and out-degree centrality measures can be calculated for both un-weighted and weighed links. In the former (binary) representation, all links are counted as one, and if a node has for example five incoming links associated to it, its in-degree centrality equals five. In the latter representation, the in- and out degree centralities equal the sum of the weights of all associated incoming and outgoing links. In this study, we calculated only the weighted in- and out-degree centralities for all nodes in the different livelihood landscapes, whereas we used both binary and weighted links for the density and centralization calculations (see below). For the binary network measures, we were mostly concern with occupational interrelations that constituted some level of significance. Thus, for these analyses we first removed all links with a weight of less than 0.05 and then set all the weights of all remaining links to unity. For all analyses taking link weights into account, no links were removed.

#### ii) Analyses of patterns of occupational interrelations

In addition to analyzing the structural position of individual occupations, we also analyzed aspects of the complete pattern of occupational interrelations in the different livelihood landscapes. Here, we focused on the structure of the broader livelihood landscape, and not on individual occupations. Two different complete pattern measures, namely network density and network centralization [28], were the focus of our analysis. The former measure captures how many (and possibly also how strong) inter-occupational dependencies there are in the livelihood landscape, and the latter captures to what extent one or a few number of occupations tends to be part of every households diverse composition of occupations. Other analytical approaches lack these complete patterns of occupational interrelations.

Network density equals, in the binary case, the number of links in the network divided by the maximum possible number of links in the network. When taking link weights into account, we summed the weights of all links and then divided that sum with the maximum possible sum of link weights in the network. The denominator is the same for both cases since the maximum possible sum of weighted links equals the maximum possible number of links (since the maximum link weight equals 1.0).

Network centralization is a measure that captures how much individual nodes differ among themselves in terms of their levels of degree centrality. It is originally defined for binary networks [28], and is constructed in such a way that the maximum value of network centralization is obtained for a perfect star network (i.e. where one star node have links to all other nodes, and the other nodes only have links to the star node)(Equation 1):
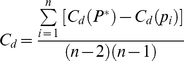
(1)C_d_ is the network centralization, C_d_(p^*^) is the maximum degree centrality among all nodes in the network, C_d_(p_i_) is the degree centrality of node _i_, and n is the total number of nodes in the network. For the weighted networks we adjusted the measure so that the degree centralities in the numerator were set to the average of each node's in- and out degree centrality. Furthermore, we multiplied the denominator with the average link weight for all links with a weight greater than zero. In that we arrived at a measure that not only consider the topology of the network, but also account for the variability of link weights in the network. We calculated both network density and network centralization for binary and weighted networks.

### Analysis: comparing network measures along spectra of socioeconomic development and human population density

In this section, we use the network measures to further explore initial findings of a “composition effect” [16] in coastal communities in the Western Indian Ocean [11]. In general, according to the composition effect, one would expect that the number of households that depend on natural resource-based occupations such as fishing and agriculture would decline with socioeconomic development [16]. One might also expect that each individual household is engaged in fewer occupations as an effect of a more pronounced division of labor and specialization in more socioeconomic developed communities. We hypothesized that the in- and out degree centralities of natural-resource occupations would decline with increasing socioeconomic development, hence implying that not only fewer households engage in such occupations, but rather that fewer households engage in these occupations while simultaneously working with something else. We also examined how population density was related to livelihood characteristics, expecting that land constraints would limit involvement in key natural resource occupations in densely populated areas [17]–[20]. We used categorical regressions with optimal scaling, employing the Lasso technique (Least Absolute Shrinkage and Selection Operator) with bootstrapping for model selection to see whether the development index or population density could better explain: a) occupational in and out-degree centrality, b) the level of network density, and c) the level of network centralization. The Lasso method allows for easy interpretation of predictor selection but is robust where independent variables exhibit multicollinearity [29]; the two independent variables were significantly correlated (Spearman's ρ = 0.51, p = 0.009). The Lasso method was used for subset selection, but subsequently the optimal model was analysed using categorical regression without shrinkage. We used Spearman's rank correlation to describe the strength of relationships between our independent variables of development and population density and binary measures of network density and centralization. Due to the exploratory nature of this paper, we also highlight (but differentiate) relationships that are significant at p<0.10.

### A note on the applied network metrics and scale

A common issue when comparing different network using many of the commonly applied network metrics such as density and centralization is that the metrics are not independent of the size of the network (see e.g. [30]). Hence, comparing metrics from two or more networks that differ significant in size (i.e. number of nodes) is often problematic. Fortunately, in our case the number of nodes in a livelihood landscape is independent on the social aggregation level (e.g. communities, regions, and nations). Instead, the size of the network is determined by the number of occupations, and if the same number of occupations is used consistently (as we did), the potential problem of comparing livelihood landscapes at varying scales is kept under control. This, we argue, is another benefit of using the suggested modeling approach to describe and analyze occupational interdependencies as networks.

### Ethics Statement

We obtained verbal consent from participants before conducting household surveys. During verbal consent, participants were informed about the survey, its purpose, and how the data would be utilized. Participant's names were not recorded. Written consent from participants was not obtained because of low literacy rates in many of our field sites, which meant that participants may not have fully understood what they signed. This project was administered by the Wildlife Conservation Society, which does not have an Institutional Review Board for research ethics regarding social science surveys.

### “Nomenclatural Acts”

The electronic version of this document does not represent a published work according to the International Code of Zoological Nomenclature (ICZN), and hence the nomenclatural acts contained in the electronic version are not available under that Code from the electronic edition. Therefore, a separate edition of this document was produced by a method that assures numerous identical and durable copies, and those copies were simultaneously obtainable (from the publication date noted on the first page of this article) for the purpose of providing a public and permanent scientific record, in accordance with Article 8.1 of the Code. The separate print-only edition is available on request from PLoS by sending a request to PLoS ONE, 185 Berry Street, Suite 3100, San Francisco, CA 94107, USA along with a check for $10 (to cover printing and postage) payable to “Public Library of Science”.

## Results and Discussion

Livelihood portfolios are critical in the context of natural resource management because the capacity to engage in multiple occupations can influence important issues such as whether fishers would exit a declining fishery [31], how fishers react to policy [32], [33], the types of management systems that may be applicable [3], [8], [34], and other natural resource-based decisions [2], [6], [19]. Consistent with other studies on rural livelihoods in coastal communities, we found that diversification was a central feature of most households' livelihood strategies [3], [6], [7]. The mean number of household occupations per community ranged from 1.1 to 2.5. In the context of this study, we were not aiming to determine the causes of livelihood diversification (e.g. [4], [6], [7], [35]), but rather provide novel insights into its pattern. This was accomplished through developing network maps of ‘livelihood landscapes’ and by examining whether systemic measures of the local economy changed predictably along spectra of socioeconomic development and population density.

### Contributing to understanding coastal livelihoods by visualizing ‘livelihood landscapes’ as networks

One goal of this paper was to develop a methodological framework to examine household-scale linkages between occupational sectors and then apply this in the context of tropical coastal communities. In order to test how and in which ways the network representation of livelihood landscapes can help in understanding and analyzing patterns of occupational interdependencies, we chose to focus this part of our analysis on different scales of social aggregation in Kenya (i.e. a single Kenyan peri-urban village, two different aggregates of several Kenyan peri-urban and rural communities respectively, and an aggregate of nine Kenyan peri-urban and rural communities). In particular, we were interested whether if these livelihood landscape maps could provide important insights into the role of key natural resource-based sectors such as fisheries in the wider economy- a critical point emphasized by other livelihood framework approaches [2], [3].

**Figure 2:**
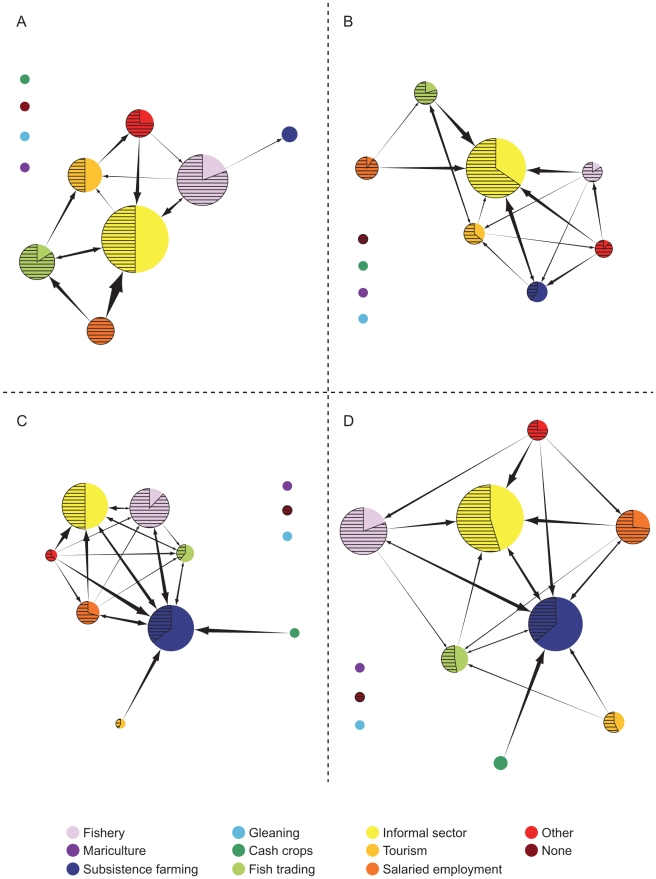
Kenyan livelihood landscape maps at various scales of social organization: a) Shela, Kenya; b) an aggregation of peri-urban sites in Kenya; c) an aggregation of rural sites in Kenya; d) all sites in Kenya. Links between occupations are indicated by arrows The size of a node indicates the relative involvement in that occupational sector (larger node means more people are involved). The direction of the arrows indicates the priority of ranking. Thus an arrow into an occupation indicates that the occupation was ranked lower than the occupation the arrow came from. The thickness of the arrows corresponds to the proportion of households being engaged in the, by themselves, higher ranked occupation that are also engaged in the lower ranked occupation. The proportion of the node that is shaded represents the proportion of people that ranked that occupation as a primary occupation.

As seen in the figure 2, the respective roles of key sectors such as fisheries can vary substantially depending on whether the scale analyzed is a single community, aggregates of rural or peri-urban areas, or an aggregate of nine coastal communities (Fig. 2). In Shela, a single peri-urban community in Kenya, the informal sector and fisheries were the two largest occupations, and the former also had the most linkages to/from other occupations (Fig. 2A). Of these links, the strongest were the ones pointing towards the node representing the informal sector (i.e. the informal sector generally ranked lower than the other occupations). In contrast, agriculture was a small node which was only linked to the rest of the network through simultaneous participation in fisheries. Furthermore, the informal sector was placed in the middle of the figure, thus its level of uniform embeddeddness was the highest in comparison to the other occupations. Hence, households engaged in the informal sector seem to have no clear propensities to engage in any other specific occupations, rather they engage uniformly in most other occupations.

When all three Kenyan peri-urban sites were aggregated, the informal sector remained the biggest and most connected node (i.e. it had most links)(Fig. 2B). However, this sector was almost entirely considered a secondary occupation (as evidenced by the incoming arrows, but only an outgoing arrow to agriculture. The other outgoing links were in this case omitted as a consequence of the 5% threshold on the link weight. i.e., we deleted linkages that involved <5% of the households in the higher ranking occupations; see method section). The high level of uniform embeddeddness of the informal sector remained during aggregation. All this suggests that in peri-urban areas of Kenya, the informal sector is a central feature of the economy, but very much a supplemental activity that households engage in irrespectively of what their main occupations happens to be. In the aggregated peri-urban sites, fishing was a small, but well-connected node mostly with outgoing linkages, suggesting that for those who fished, it was a relatively important occupation. Its position in the figure is a bit more in the periphery as compared to the informal sector. This is a consequence of the lack of links between fishing and the sectors of employed salaries and selling of marine products, i.e. households engaged in fishing did not simultaneously engage in these other sectors. Thus, compared with the informal sector, fishing was less uniformly connected to the other sectors at this aggregated community level. Furthermore, the figure illustrate how the sectors of salaried employment and the selling of marine products differentiated themselves, as a group, from the other occupations by their placement on the left hand side of the network. On the other hand, fishing together with tourism, agriculture and the sector of other occupations grouped together on the right hand side of the network, thus illustrating how these sectors are roughly equivalent in terms of their relations among themselves and to others.

In rural areas, natural resource based nodes, such as fisheries and agriculture were large and thus engaged in by a large number of households (Fig. 2C). Agriculture was the most connected node in rural sites, with both incoming and outgoing links between most sectors (except cash crops and tourism, which only had outgoing links to agriculture). Furthermore, agriculture was, in contrast to the peri-urban community aggregate, the most uniformly embedded sector, whereas the informal sector is more in the periphery. In rural communities, the fisheries sector was a large and well-connected node. Interestingly there were more linkages between fisheries and other sectors in the rural aggregate than in other social configurations. A weak connection between salaried employment and fisheries is evident in the rural community aggregate, but not in other configurations. Thus, in rural communities, some households engaging in salaried employment also fish.

When both peri-urban and rural communities were aggregated, the resulting network map shows how the different tendencies in peri-urban and rural communities sum up (Fig. 2d). For example, both the informal sector and agriculture occupies positions in the middle of the figure (i.e. they are both approximately equally uniformly embedded). Hence, the aggregation of all communities does not distort the patterns revealed above, but rather these patterns are integrated to allow for analyses at larger scales. However, the earlier smaller-scale analyses showed how characteristics of the livelihood landscapes differed between peri-urban and rural communities, thus these smaller-scale figures helped to unpack the pattern of occupational dependencies seen on the more aggregated level.

We conclude this subsection by arguing that this rather qualitative analysis of the mapped-out livelihood landscape allows donors, managers, and policy makers to visualize considerable information about participation in and the relationship between occupations in one figure, which would normally require simultaneously interpreting several tables and figures. Specifically, network maps include information about participation (the size of the node), the level of uniformly embeddeddness of the sector (locations are based on spring-embedding techniques), the level of occupational primacy (the shaded proportion of the node correspond the fraction of household reporting this occupation as their primary source of income), whether households are simultaneously engaged in sectors (arrows between the nodes), the relative importance of sectors (the directionality of arrows), and the strength of interrelationships between sectors (the width of arrows) (Fig 2).

### Relationships between quantitative livelihood landscape characteristics, socioeconomic development, and human population density

#### i) Degree centralities of different occupations

Here, we examined whether aspects of dependence on natural resource-based occupations changed predictably with socioeconomic development or population density. Our results were broadly supportive of both a composition effect and the notion that population density may structure aspects of livelihood landscapes across the WIO [17]–[20]. While both population density and socioeconomic development predicted some livelihood landscape characteristics, there were more statistically significant relationships associated with socioeconomic development (Table 1), particularly when household size was accounted for (Table S1).

**Table 1:** Relationships between network statistics, socioeconomic development and population density not accounting for household sizes.

Network statistic	Development Beta	Population (ln) Beta	F	r^2^	p
Fishing out-degree	−0.63	NA	16.21	0.39	<.001
Fishing in-degree	NA	−0.40	4.34	0.16	0.048
Selling marine products out-degree	−0.55	NA	10.68	0.30	0.003
Selling marine products in-degree	−0.55	NA	13.67	0.35	0.001
Farming out-degree	−0.33	NA	3.10	0.11	0.091
Farming in-degree	−0.73	NA	27.72	0.53	<.001
Cash crops out-degree	NA	−0.33	2.62	0.11	0.12
Cash crops in-degree	−0.29	−0.38	5.47	0.33	0.012
Salaried out-degree	NA	−0.48	6.95	0.23	0.015
Salaried in-degree	0.28	NA	2.12	0.08	0.164
Tourism out-degree	NA	−0.32	2.56	0.10	0.123
Tourism in-degree	0.43	NA	5.69	0.19	0.025
Informal out-degree	NA	−0.51	7.93	0.26	0.01
Informal in-degree	−0.36	0.36	1.66	0.13	0.213
Density	−0.37	−0.33	6.27	0.36	0.007
Centralization	−0.58	NA	12.47	0.33	0.002

Results are from categorical regression analysis. Bold denotes a relationship significant at α<0.05. Italics denotes a relationship significant at α<0.10.

**Figure 3:**
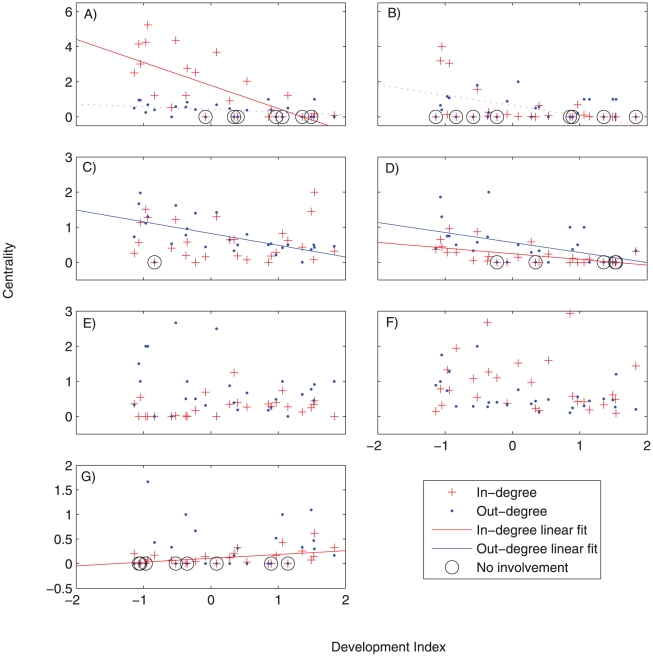
Relationships between a multi-variate index of socioeconomic development (x-axis) and centrality measures for key occupational sectors. a) agriculture; b) cash crops; c) fish; d) fish trader/middle man, e salaried employment; f) informal economy, g) tourism. Significant relationships are indicated with trend lines (P<0.05, see Table 1). Dotted lines represent relationships where p<0.10. If the population density was a better predictor than socioeconomic development, or if the relationship is not significant, no trend lines are drawn. Note that 0 centrality due to no involvement in the occupation is distinguished from 0 centrality due to no incoming or outgoing links by a black circle surrounding the former; both were included in the regression analysis.

**Figure 4:**
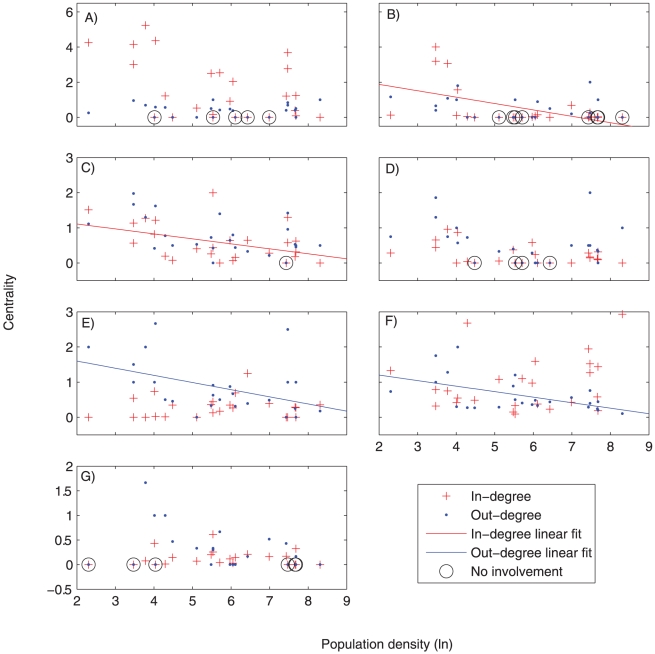
Relationships between population density (x-axis) and centrality measures for key occupational sectors. a) agriculture; b) cash crops; c) fish; d) fish trader/middle man, e salaried employment; f) informal economy, g) tourism. Significant relationships are indicated with trend lines (P<0.05, see Table 1). Dotted lines represent relationships where p<0.10. If the development index was a better predictor than population density, or if the relationship is not significant, no trend lines are drawn. Note that 0 centrality due to no involvement in the occupation is distinguished from 0 centrality due to no incoming or outgoing links by a black circle surrounding the former; both were included in the regression analysis.

Most natural resource based occupations (including fishing, farming, and selling marine products) tended to lose centrality with either development or population density. Importantly, relationships between the different types of centrality (in-degree and out-degree) and both development and population density varied by sector (Table 1, Fig. 3, Fig. 4). Figures 3 and 4 complement Table 1 by illustrating how the degree centralities change along the socioeconomic development and population density spectra.

The fishery (both capture fishery and fish trading) experienced declining in/out-degree centrality with development (Table 1, Fig 3). For example, the tendency to do something less important than fishing declined significantly with development, whereas the tendency to do fishing as a less important activity (as reflected by the in-degree centrality) did not significantly change with development. It is important to note that in and out-degree centrality does not necessarily measure primary versus secondary occupations, but rather relative rankings. Put another way, fishing households in less developed communities were likely to supplement fishing, whereas fishers in a wealthier community were not as likely to have an occupation less important than fishing. This may represent increasing professionalization of fisheries so that households which rely on fisheries had less supplementary occupations at higher levels of development. Meanwhile, fisheries as a supplemental occupation did not change significantly with development, partly due to recreational fishing in highly developed places such as Seychelles. However, the role of fishing as a supplemental livelihood did decline with population density.

Alternatively the in-degree centrality in the farming sector displayed a significant decline with development (Table 1). Thus, the tendency to combine farming with a more important occupation decreased with development. This is consistent with findings from Ivory Coast and western Kenya, which found that the wealthy bifurcate into two groups- full-time farmers and those that don't farm [4], [36]. Our expectation was that farming would be highly sensitive to land constraints, but interestingly neither in- or out-degree centrality of the farming sector were predicted by population density. One explanation for this may be that our grouping was too coarse and that there may be considerable variation in the types of agricultural production within the agricultural sector along the gradient of population density [e.g. 17]. Accordingly, the in-degree centrality of cash crops declined with population density (and development remained in the regression model as marginally significant). Thus, the tendency to engage in the cash crop sector as a supplemental occupation declined with population density and development.

Neither salaried employment nor the informal sector were significantly influenced by development, but did change along gradients of population density. Our initial assumption was that the salaried employment sector would have increased in relative importance with development. Although not showed here, the number of households engaged in salaried employment generally increased with development, but the extent to which households engaged in salaried employment also engage in other occupations did not vary significantly. However, population density appeared to influence whether households relying on salaried employment or the informal sector engage in supplemental activities. Specifically, the out-degree centrality of salaried employment and the informal sector declined significantly with population density. This means that households in densely populated areas which rely on salaried employment or informal activities are less likely to have supplementary occupations than similar households in less densely populated places.

Only selling marine products displayed significant declines in both in- and out-degree centrality with development. As the level of development increased, trading fish was less likely to be combined with any other occupations – this is engaging in specialization. This is in contrast to the capture fishery sector, which (as said above) is maintained as a less-important occupation at higher levels of development, likely due to the increasing prevalence of recreational fishing in high development areas.

Most sectors showed either a significant decrease in in- and/or out degree centrality, or remained unaffected, with development or population density. The exception to this was the tourism sector which showed a positive relationship between development and in-degree centrality. Thus, with development, multiple-occupation households engaged in tourism are increasingly likely to have other, more important occupations. In the more developed communities, working in tourism may largely be a secondary livelihood strategy because tourism-related jobs may fail to provide adequate income or stability to meet household needs.

#### ii) System-level analyses of connectivity in livelihood landscapes

In addition to the in and out-degree measures of centrality, we also examined system-level measures of connectivity among all sectors within a community to provide insights into the role of household specialization or diversification with development. There was a significant negative relationship between community development and the mean number of occupations per household (Spearman's ρ = −0.41, p = 0.03). However, this correlation does not provide information about how the overall pattern of occupational interrelationships in the local economy changes. The measure of network density, on the other hand, captures how many different occupational combinations there exist among the households in the studied community. Overall, there was a decrease in the level of weighted network centralization as socioeconomic development increased (Table 1). Likewise, the weighted network density declined as socioeconomic development and also population density increased (Table 1). For weighted network density, neither term in the model was significant, but the overall model was.

From a topological perspective (i.e. when disregarding link weights), a high network density tells us that there are a high number of occupations per household, but also that these occupations differ between households. In other words, if all households engaged in the same set of occupations, the un-weighted network density would typically remain low. Low frequencies in some binary categories made categorical regressions unreliable, so for binary density and centralization measures, we used Spearman's correlations. Binary network density was related to population density (Spearman's ρ = −0.6, p = 0.001) and to development (Spearman's ρ = −0.33, p = 0.046). Thus, the un-weighted network density decreased predictably with both development and population density. This means that in low development and low population sites, households had a higher number of different occupations, but also that any individual occupation was more likely to be linked to several other occupations by households engaged in both. In this context it should be pointed out that when taking link weight into account, the network density measure itself approaches the simpler measure of number of occupation per households (i.e., any diversity in terms of different sets of occupations per households is cancelled out). Hence, the binary and the weighted network density measures each captures different characteristics of the livelihood landscapes.

Both weighted and binary measures of centralization declined predictably with development (Table 1, Spearman's ρ = −.065, p<0.001, respectively). However, neither centralization measure changed with population density (Table 1, Spearman's ρ = −.027, p<0.18, respectively). These results were virtually identical when household size was accounted for (Table S1). This means that in low development communities, a relatively higher proportion of the households were engaged in at least one common sector compared to more developed communities. In other words, in more developed communities, the tendency for many multiple-occupation households to have a common sector was less pronounced. The level of centralization is not completely detached from the density measure because very low or very high densities are unlikely to be accompanied by high levels of centralization. However, these two measures provide unique information about the configuration of livelihood landscapes.

Altogether, both the dense networks and high level of centralization associated with lower development and lower population density may impact how communities approach natural resource debates. In particular, high occupational network density may mean that more households act as links between sectors that may have conflicting incentives regarding resource management. For example, high density networks are more likely to have households with someone from the fishery sector (who, for example, may oppose a proposed marine protected area that will limit fishing) and someone working in the tourism sector (who may benefit from the proposed marine protected area due to increased tourism). As livelihood landscapes become less dense, some occupations are likely to have weaker links to other occupations. In this context, the activities of households and communities are likely to become siloed and people are less likely to be knowledgeable about, and potentially sympathetic to, the positions of others. Additionally, as centralization declines, communities may also lose the cohesion and social interaction that may be generated from having at least one common sector. Communities that have highly specialized local economies may lack ‘brokering’ households, and consequently conflicts over resource allocation may become more entrenched. This is not to suggest that there are few conflicts over natural resources in low development sites, but rather that peoples' perspectives and approach to conflict resolution may be different because they are more likely to have a shared understanding.

Our initial investigation of how natural resource sectors change along spectra of socioeconomic development and population density was a first step in providing novel insights into observed patterns [11], but has some shortcomings that could potentially be addressed in future studies of livelihood landscapes. First, our study only examines a limited number of potential variables to explain livelihood landscapes. Other alternative models might better explain livelihood landscape patterns (e.g. kinship networks, tenure arrangements, entitlements to resources, and incomplete markets for land, labor, or credit; [4], [37]–[39]). For example, studies have found that land tenure and caste systems influence livelihood diversity [40], [41]. Secondly, our paper does not attempt to unravel the complicated social consequences of development and livelihood diversification. Issues of equity, local aspirations, gender, and power relations have long been central to many development and livelihood studies (e.g., [38], [42]–[44]), yet were beyond the scope of this present paper. These issues are critical because they, along with global politics [45], can shape or constrain development pathways, with potentially severe consequences for both societies and ecosystems. For example, critical reviews of the role of development in fisheries suggest that development policies such as structural adjustment programs (particularly market and political liberalization, macroeconomic reforms, and decentralization) resulted in a dismantling of critical governance institutions, a lack of support for new institutions, and a subsequent ecological crisis in many developing country fisheries [46]. Additionally, it is important to note that our observed changing patterns of local resource use do not necessarily mean that societies with higher levels of development are more ‘environmentally friendly’. Despite the potential for some local-scale environmental conditions to improve with development, wealthier societies tend to consume more and often able to garner resources from further afield [47]. Thus, wealthier societies often impacts ecosystems at larger scales [11], [12], [47], [48].

### Conclusion

The combination of sectoral and systemic network analyses suggest an increase in specialization with development for most sectors, including fishing and farming, but at the community level, economies remain diversified. This apparent professionalization of natural-resource-based occupations along the spectrum of development has some implications for natural resource use and management. At the household scale, diverse livelihood portfolios are generally seen as a source of resilience in the face of adverse trends or sudden shocks [2], [3], [49], [50]. For example, in Tanjona, Madagascar, some local residents responded a collapse of vanilla prices by increasing effort in the fishery [51]. A reduction of household livelihood diversity may erode aspects of society's capacity to deal with change (often referred to as adaptive capacity) relating to flexibility, while development may foster other aspects related to access to crucial assets that may help people weather disturbances [51]. A key question for future analyses is how the lack of household diversity, but maintenance of community diversity we see with increasing development influences different aspects of vulnerability to environmental change or disasters. Critical to this may be the increasing involvement (albeit secondary) in the tourism sector, which can be subject to severe global shocks and create new vulnerabilities [52] and the role of social insurance, which can substitute for the self-insurance of livelihood diversification [4].

Finally, this novel approach to examining livelihood landscapes complements existing livelihood frameworks by providing a new way to visualize, in a compact and comprehensive format, complicated patterns of interrelationships between livelihoods. Furthermore, these livelihood landscapes also provide for quantitative system-level investigations, utilizing network analytical approaches, about patterns of inter-relationships between occupations that have not been previously examined. This network-based approach to livelihood landscapes is broadly applicable to understanding livelihoods in other social-ecological systems. Future applications could compare livelihood landscapes between groups (e.g. migrants and non-migrants), explore how these networks vary along tenure institutions or kinship-networks, examine shared tasks in the workplace, and potentially incorporate other network statistics such as structural equivalents to determine potential substitutability between occupations. In this paper, we have just scratched the surface on what we foresee as being a potentially very useful and easily extendable research approach in furthering our understanding on how livelihood strategies influence how natural resources are used and misused by societies at varying levels of social aggregation, ranging from remote villages to nations and beyond. In particular, we believe that further studies making use of numerous other metrics and analyses developed within the broad interdisciplinary field of network analysis can contribute with new insights on how different patterns of relations in livelihood landscapes relates to various aspects of socioeconomic development.

## Supporting Information

Table S1 Relationships between network statistics, socioeconomic development and population density with household size accounted for. Results are from categorical regression analysis. Bold denotes a relationship significant at α<0.05. Italics denotes a relationship significant at α<0.10. The models that did and did not account for household size were generally similar, although several marginally significant relationships became significant at α<0.05 when household size was accounted for (Table 1). Specifically, out-degree centrality of farming, and network density were significant when household size was accounted for, while in-degree centrality of tourism was not significant when household size was considered. Additionally, informal out-degree centrality of the informal sector was significant when accounting for household size.
(0.04 MB DOC)

## References

[1] Cinner J, McClanahan T, Wamukota A (2010). Differences in livelihoods, socioeconomic characteristics, and environmental perceptions between fishers and non-fishers living near and far from marine parks on the Kenyan coast.. Marine Policy.

[2] Pomeroy R, Ratner B, Hall S, Pimoljinda J, Vivekanandan V (2006). Coping with disaster: Rehabilitating coastal livelihoods and communities.. Marine Policy.

[3] Allison E, Ellis F (2001). The livelihoods approach and management of small-scale fisheries.. Marine Policy.

[4] Barrett CB, Reardon T, Webb P (2001). Nonfarm income diversification and household livelihood strategies in rural Africa: concepts, dynamics and policy implications.. Food Policy.

[5] Rigg J (1998). Rural-urban interactions, agriculture and wealth: a southeast Asian perspective.. Progress in Human Geography.

[6] Ellis F (1999). Household strategies and rural livelihood diversification.. Journal of development studies.

[7] Ellis F (2000). The determinants of rural livelihood diversification in developing countries.. Journal of Agricultural Economics.

[8] Salafsky N, Wollenberg E (2000). Linking livelihoods and conservation: A conceptual framework and scale for assessing the integration of human needs and biodiversity.. World Development.

[9] Borgatti S, Mehra A, Brass D, Labianca G (2009). Network analysis in the social sciences.. Science.

[10] Bodin O, Crona B (2009). The role of social networks in natural resource governance: What relational patterns make a difference?. Global Environmental Change.

[11] Cinner J, McClanahan T, Daw T, Graham N, Maina J (2009). Linking social and ecological systems to sustain coral reef fisheries.. Curr Biol.

[12] York R, Rosa E, Dietz T (2003). Footprints on the earth: the environmental consequence of modernity.. American Sociological Review.

[13] Clausen R, York R (2008). Economic growth and marine biodiversity: influence of human social structure on decline of marine trophic levels.. Conservation Biology.

[14] Clausen R, York R (2008). Global biodiversity decline of marine and freshwater fish: a cross-national analysis of economic, demographic, and ecological influences.. Social Science Research.

[15] Grossman G, Krueger A (1995). Economic growth and the environment.. Quarterly Journal of Economics.

[16] Grossman G, Krueger A (1991). Environmental impacts of the North American free trade agreement.. NBER.

[17] Boserup E (1965). The conditions of agricultural growth: the economics of agrarian change under population pressure.

[18] Pender J (2004). Development pathways for hillsides and highlands: some lessons from central America and east Africa.. Food Policy.

[19] Koczberski G, Curry G (2005). Making a living: land pressures and changing livelihood strategies among oil palm settlers in Papua New Guinea.. Agricultural Systems.

[20] Jansen H, Rodriguez A, Damon A, Pender J, Chenier J (2006). Determinants of income-earning strategies and adoption of conservation practices in hillside communities in rural Honduras.. Agricultural Systems.

[21] McClanahan TR, Cinner J, Maina J, Graham N, Daw T (2008). Conservation action in a changing climate.. Conservation Letters.

[22] McClanahan TR, Cinner J, Maina J, Graham N, Daw T (2009). Identifying reefs of hope and hopeful actions: Contextualizing environmental, ecological, and social parameters to effectively respond to climate change.. Conservation Biology.

[23] Henry G (1990). Practical sampling.

[24] Pollnac R (1998). Rapid assessment of management parameters for coral reefs.

[25] Degenne A, Forse' M (1999). Introducing social networks.

[26] Borgatti S, Everett M, Freeman L (2002). Ucinet for Windows: software for social network analysis.. Harvard Analytic Technologies 2006.

[27] Wasserman S, Faust K (1994). Social network analysis: Methods and applications.

[28] Freeman L (1979). Centrality in social networks. Conceptual clarifications.. Social Networks.

[29] van der Kooij AJ (2007). Prediction accuracy and stability of regression with optimal scaling transformations: the .632 bootstrap with CATREG.

[30] Butts CT (2006). Exact bounds for degree centralization.. Social Networks.

[31] Cinner J, Daw T, McClanahan T (2009). Socioeconomic factors that affect artisanal fishers' readiness to exit a declining fishery.. Conservation Biology.

[32] Marshall N, Fenton D, Marshall P, Sutton S (2007). How resource-dependency can influence social resilience within a primary resource industry.. Rural Sociology.

[33] Marshall N, Marshall P (2007). Conceptualizing and operationalizing social resilience within commercial fisheries in northern Australia.. Ecology & Society.

[34] Cinner J (2007). Designing marine reserves to reflect local socioeconomic conditions: lessons from long-enduring customary management systems.. Coral Reefs.

[35] Abdulai A, CroleRees A (2001). Determinants of income diversification amongst rural households in Southern Mali.. Food Policy.

[36] Barrett CB, Bezuneh M, Aboud A (2001). Income diversification, poverty traps and policy shocks in Cote d'Ivoire and Kenya.. Food Policy.

[37] Sen A (1981). Ingredients of famine analysis: availability and entitlements.. Quaterly Journal of Economics.

[38] Leach M, Mearns R, Scoones I (1999). Environmental entitlements: dynamics and institutions in community-based natural resource management.. World Development.

[39] Carrier J, Carrier AH (1989). Wage, trade and exchange in Melanesia: a Manus society in the modern state.

[40] Start D, Deshingkar P, Farrington J, Nayyar R, Sharma AN (2005). Influence of wealth on patterns of livelihood diversification: Evidence from field studies in aNdhra Pradesh and Madhya Pradesh.. Rural transformation in India: the role of non-farm sector.

[41] Coulthard S (2008). Adapting to environmental change in artisanal fisheries - insight from a South Indian lagoon.. Global Environmental Change.

[42] Kuznets S (1955). Economic growth and income inequality.. American Economic Review.

[43] Vandergeest P, Buttel FH (1988). Marx, Weber, and development sociology: Beyond the impasse.. World Development.

[44] Niehof A (2004). The significance of diversification for rural livelihood systems.. Food Policy.

[45] Chirot D, Hall TD (1982). World-system theory.. Annual Review of Sociology.

[46] Bene C, Lawton R, Allison EH (2010). Trade matters in the fight against poverty: Narratives, perceptions, and (lack of) evidence in the case of fish trade in Africa.. World Development.

[47] Arrow K, Bolin B, Costanza R, Dasgupta P, Holling CS (1995). Economic growth, carrying capacity and the environment.. Science.

[48] Berkes F, Seixas C (2006). Building resilience in lagoon social-ecological systems: a local-level perspective.. Ecosystems.

[49] Campbell D (1990). Strategies for coping with severe food deficits in rural Africa: a review of the literature.. Food and Foodways.

[50] Carter M (1997). Environment, technology and the social articulation of risk in West African agriculture.. Economic Development and Cultural Change.

[51] Cinner J, Fuentes M, Randriamahazo H (2009). Exploring social resilience in Madagascar's marine protected areas.. Ecology & Society.

[52] Gossling S, Hall M (2006). Tourism & global environmental change - Ecological, social, economic and political interrelationships.

