# Supplementary material for: Livelihood Diversification in Tropical Coastal Communities: A Network-Based Approach to Analyzing ‘Livelihood Landscapes’
Source: PLoS One. 2010 Aug 11;5(8):e11999. doi: 10.1371/journal.pone.0011999 (PMC2920305; doi:10.1371/journal.pone.0011999)
Supplement: Table S1 — Relationships between network statistics, socioeconomic development and population density with household size accounted for. Results are from categorical regression analysis. Bold denotes a relationship significant at α<0.05. Italics denotes a relationship significant at α<0.10. The models that did and did not account for household size were generally similar, although several marginally significant relationships became significant at α<0.05 when household size was accounted for (Table 1). Specifically, out-degree centrality of farming, and network density were significant when household size was accounted for, while in-degree centrality of tourism was not significant when household size was considered. Additionally, informal out-degree centrality of the informal sector was significant when accounting for household size. (0.04 MB DOC) [file pone.0011999.s001.doc]

| **Network statistic** | **Development Beta** | **Population density (ln) Beta** | **F** | **r^2^** | **p** |
| --- | --- | --- | --- | --- | --- |
| Fishing out-degree | **-0.70** | NA | 23.65 | 0.49 | **<.001** |
| Fishing in-degree | NA | **-0.49** | 7.44 | 0.24 | **0.012** |
| Selling marine products out-degree | **-0.60** | NA | 14.03 | 0.36 | **0.001** |
| Selling marine products in-degree | **-0.64** | NA | 30.17 | 0.41 | **<.001** |
| Farming out-degree | **-0.42** | NA | 5.30 | 0.18 | **0.03** |
| Farming in-degree | **-0.78** | NA | 38.61 | 0.61 | **<.001** |
| Cash crops out-degree | NA | -0.36 | 3.44 | 0.13 | 0.076 |
| Cash crops in-degree | -0.29 | **-0.38** | 5.52 | 0.33 | **0.011** |
| Salaried out-degree | NA | **-0.51** | 8.25 | 0.26 | **0.009** |
| Salaried in-degree | 0.19 | NA | 0.95 | 0.04 | 0.357 |
| Tourism out-degree | NA | -0.28 | 1.93 | 0.08 | 0.178 |
| Tourism in-degree | 0.35 | NA | 3.44 | 0.12 | *0.075* |
| Informal out-degree | **-0.41** | -0.29 | 6.34 | 0.37 | **0.007** |
| Informal in-degree | -0.35 | NA | 3.47 | 0.12 | *0.074* |
| Density | **-0.51** | -0.27 | 9.89 | 0.47 | **0.001** |
| Centralization | **-0.49** | NA | 7.86 | 0.24 | **0.01** |

Table S1. Relationships between network statistics, socioeconomic development and population density with household size accounted for. Results are from categorical regression analysis. Bold denotes a relationship significant at α<0.05. Italics denotes a relationship significant at α<0.10. The models that did and did not account for household size were generally similar, although several marginally significant relationships became significant at α<0.05 when household size was accounted for (Table 1). Specifically, out-degree centrality of farming, and network density were significant when household size was accounted for, while in-degree centrality of tourism was not significant when household size was considered. Additionally, informal out-degree centrality of the informal sector was significant when accounting for household size.
